# Research on Tunable Ultraviolet Detector and Photoresponse Mechanism Based on In:Ga_2_O_3_/p-GaN Heterojunction

**DOI:** 10.3390/s26041197

**Published:** 2026-02-12

**Authors:** Xiang Wang, Xiao Wang, Ping Zhang, Yun Li, Xiaohuai Wang, Youming Lu

**Affiliations:** 1 Department of Physics and Electronic Engineering, Hanshan Normal University, Chaozhou 521041, China; xwang@hstc.edu.cn (X.W.); 20220052@hstc.edu.cn (P.Z.); liyunphy@hstc.edu.cn (Y.L.); 2 College of Materials Science and Engineering, Shenzhen University, Shenzhen 518060, China; wangxiaoszu@163.com

**Keywords:** ultraviolet photodetector, In:Ga_2_O_3_/p-GaN heterojunction, tunable photoresponse

## Abstract

The ultraviolet photodetectors based on In:Ga_2_O_3_/p-GaN heterojunctions were fabricated by depositing an In:Ga_2_O_3_ thin film on a p-GaN substrate under different oxygen pressures using the pulsed laser deposition method. The devices exhibit typical self-powered behavior and a broad-spectrum response within the wavelength range of 250–345 nm. Under low oxygen pressure, the self-powered response peak of photodetectors with negative response current is mainly located at 345 nm, corresponding to the p-GaN layer. When the oxygen pressure exceeds 5 Pa, the response peak at 250 nm related to the In:Ga_2_O_3_ layer becomes the predominant peak, and the response current is positive. Studies demonstrate that the response peaks at 345 nm and 250 nm of the devices could be modulated by varying the applied bias voltage. The results indicate that, as the reverse bias increases, the response peak in the near ultraviolet region gradually decreases, while the response peak in the solar blind ultraviolet region gradually increases. The tunable photoresponse mechanism is attributed to the changes in the spatial-charge region and built-in electric field caused by devices prepared under different oxygen pressures and by varying the reverse bias applied to the devices.

## 1. Introduction

Recently, ultraviolet (UV) photodetectors (PDs) have garnered significant interest due to their wide-ranging applications, including monitoring the ozone layer, detecting flames, facilitating space communication, enhancing military surveillance, enabling biochemical detection, and inspecting for UV leaks [1,2,3,4,5]. Due to the complexity of the application environment, single-band detectors are increasingly finding it difficult to meet the needs of practical applications. Compared to highly selective single-spectrum PDs, PDs with a wider range of UV responses exhibit enhanced environmental adaptability and anti-jamming capabilities, leading to a significant reduction in false alarm rates and an improvement in detection accuracy. Consequently, the monolithic integration technology for broadband or multi-band detection has emerged as a focal point of innovation and a developmental research topic [6,7,8,9,10]. In order to achieve efficient multi-band detection, the most commonly used method is to use two different materials to construct heterojunction diodes [11,12,13,14,15]. Wide bandgap semiconductor materials, including gallium oxide (Ga_2_O_3_) [16,17], zinc oxide (ZnO) [18], aluminum nitride (AlN) [19], aluminum gallium nitrogen (AlGaN) [20], gallium nitride (GaN) [21], and diamond [22], are ideal for preparing high efficiency ultraviolet PDs due to their wide bandgap and high material stability. In particular, Ga_2_O_3_ has emerged as a leading and promising semiconductor material for solar-blind UV photodetectors (SBPDs) in recent years, owing to its advantageous properties such as a suitable bandgap, high electron mobility, and high breakdown voltage. These characteristics enable Ga_2_O_3_ to effectively detect solar-blind UV radiation, making it a material of choice for researchers in the field. Despite the challenges, Ga_2_O_3_-based SBPDs have demonstrated significant potential in terms of sensitivity, response speed, and stability, as evidenced by recent studies [23,24,25,26,27]. Although Ga_2_O_3_ demonstrates an intrinsic n-type semiconductor property due to oxygen deficiency, it presents low electron concentration and high resistivity, which hinders the effective collection of photo-generated carriers. The lower electron concentration leads to a Fermi level close to the middle of the energy band, which reduces the built-in barrier of the p–n junction and is not conducive to the separation of photo-generated carriers. Element doping has been introduced to reduce the resistance of Ga_2_O_3_ and realize n-type conductivity. Many elements, such as Ti, Zn, Sn, and In, were used to dope the Ga_2_O_3_ material [28,29,30,31,32]. PDs based on doped Ga_2_O_3_ have a significant improvement in performance. Another disadvantage of Ga_2_O_3_ is the difficulty of p-type doping, primarily due to the substantial difficulties in incorporating acceptor impurities and generating holes within the material. Theoretical calculation results indicate that the activation energy for acceptors in Ga_2_O_3_ is higher than 1 eV. Moreover, its flat valence band results in a large effective hole mass, low hole mobility, and a low diffusion constant. To date, the realization of p-type doping in Ga_2_O_3_ has been a significant challenge, hindering the development of p–n junctions, UV PDs, and bipolar transistors. Therefore, it is common to choose other p-type semiconductors or n-type semiconductors with large band offsets to construct p–n or n–n Ga_2_O_3_ heterojunctions as an alternative method. GaN, with its bandgap of approximately 3.4 eV, is capable of covering the UV-to-SBUV wavelength range. Moreover, a p-type GaN thin film layer can be readily obtained by doping Mg or Si. The combination of Ga_2_O_3_ and p-GaN to form a heterojunction can effectively integrate the advantages of the two materials, thereby making up for their respective deficiencies in UV broadband detection and providing multi-band detection capabilities [6,7,8]. The low lattice mismatch and small conduction band shift at the Ga_2_O_3_/p-GaN interface suggest that this heterojunction has great potential for application in UV PDs. Consequently, the construction of self-powered photovoltaic PDs based on the Ga_2_O_3_/p-GaN heterojunction has been attracting increasing attention, and many research results on Ga_2_O_3_/p-GaN heterojunctions have been reported [4,6,7,30]. However, the construction of Ga_2_O_3_/p-GaN heterojunctions still faces some challenges. For UV PDs, the photoresponsivities are still low because of the high resistivity in the Ga_2_O_3_ layer. Spectral response range is relatively narrow, and it is inconvenient to adjust the detection wavelength of a high-selectivity spectral response device. In view of these shortcomings, alloying engineering through doping is gradually becoming one research emphasis. Indium and gallium belong to group III, with similar electronic structures and comparable atomic radii. Hence, doping indium atoms into Ga_2_O_3_ is the most suitable bandgap tailoring method used, with minimal damage to the crystal quality. There have been some studies on the (In_x_Ga_1−x_)_2_O_3_ thin film-based UV photodetectors [16,33,34,35,36,37], which can modulate the electronic structure of Ga_2_O_3_ via incorporation of In to achieve high-performance optoelectronic devices. UV photodetectors based on In-doped Ga_2_O_3_ show better electrical properties and a larger built-in potential barrier, which improves device performance [16]. The bandgap of (In_x_Ga_1−x_)_2_O_3_ alloying thin film can be modulated by controlling the In content in the alloying films, which can improve the spectral response range and achieve a tunable spectral response [35,36]. Furthermore, doping with In can introduce more intrinsic defects into Ga_2_O_3_, which act as donors to generate free electrons, thereby improving the poor conductivity of Ga_2_O_3_ [37]. However, high indium content in the (In_x_Ga_1−x_)_2_O_3_ films causes phase separation and large dark current, which is not conducive to device performance. The influence of In content on PDs’ performance and spectral response mechanism needs to be further studied.

In this study, we prepared a series of PDs based on the In:Ga_2_O_3_/p-GaN heterojunctions using a pulsed laser deposition (PLD) method with different deposition oxygen pressures. With an increase in oxygen pressure, the indium content within the film decreases, leading to a reduction in carrier concentration and a subsequent rise in resistance. The crystallinity and optical and conductivity properties are also affected by oxygen pressure. Based on these results, the spectral response of In:Ga_2_O_3_/p-GaN heterojunction PDs under zero bias shows tunable characteristics by changing the oxygen pressure. With the increase of oxygen pressure, the response peaks of In:Ga_2_O_3_ increase gradually, while the peaks related to GaN decrease gradually. The response characteristics of the devices under different reverse bias voltages were also investigated. The results indicate that, as the reverse bias increases to −2.5 V, the response peak in the near ultraviolet region gradually decreases, while that in the solar blind ultraviolet region gradually increases. The tunable photoresponse spectra effect is attributed to the changes in the spatial-charge region and built-in electric field caused by oxygen pressure and reverse bias applied to the device. The tunable ultraviolet PDs operating in the solar-blind band and near-ultraviolet band can be achieved in the In:Ga_2_O_3_/p-GaN heterojunction. This research offers a method for developing tunable photoresponse peak UV PDs or optoelectronic applications.

## 2. Materials and Methods

**Figure 1:**
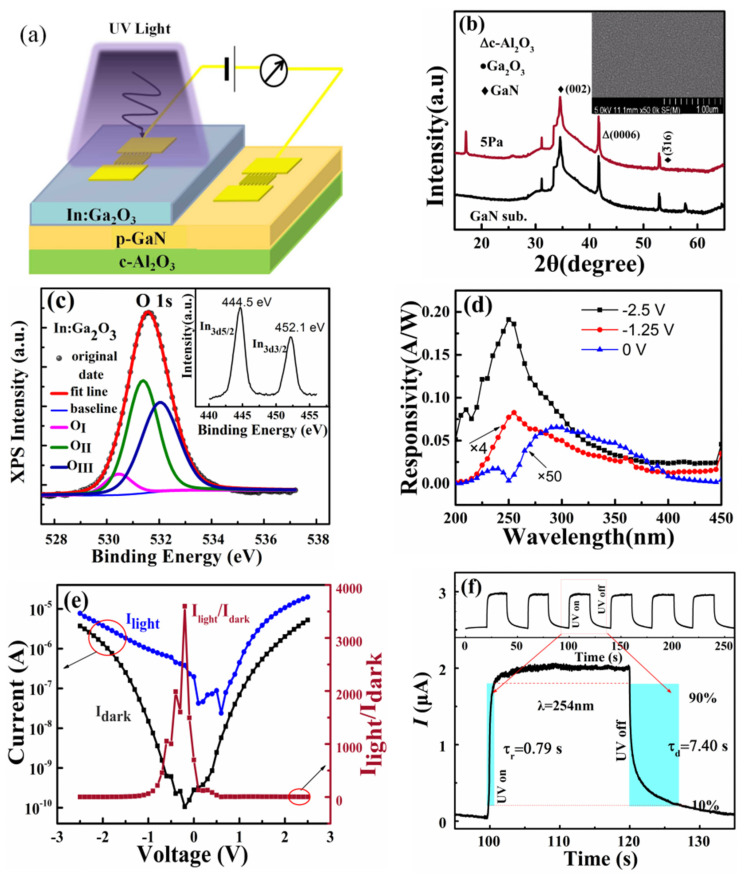
(**a**) Schematic illustration of the In:Ga_2_O_3_/p-GaN heterojunction. (**b**) XRD patterns of In:Ga_2_O_3_/p-GaN heterojunction deposited with oxygen pressure of 5 Pa. The inset is plane SEM image of In:Ga_2_O_3_/p-GaN heterojunction. (**c**) XPS spectra of O 1s from In:Ga_2_O_3_ films. The inset is the XPS spectra of In 3d. (**d**) The responsivity of In:Ga_2_O_3_/p-GaN heterojunction deposited with oxygen pressure of 5 Pa under different reverse bias. (**e**) The I–V curves of In:Ga_2_O_3_/p-GaN heterojunction. (**f**) The time-dependent photoresponse of In:Ga_2_O_3_/p-GaN heterojunction device.

In this work, In:Ga_2_O_3_ thin films were prepared on c-Al_2_O_3_ substrates and p-GaN substrates, respectively, using a pulsed laser deposition (PLD) method. The sample deposited on the c-Al_2_O_3_ substrate was used to characterize the optical properties and microstructure of the In:Ga_2_O_3_ films, while the sample deposited on the p-GaN substrate was used to fabricate the UV photodetectors. Prior to deposition, the substrates were sequentially cleaned with acetone solution (CH_3_COCH_3_) and methanol solution (CH_3_OH) to remove organic substances and impurities, adsorbed on the surface of the substrates. Then, diluted hydrofluoric acid solution (HF:H_2_O = 1:10) was used to remove the natural oxide layer on p-GaN surface. An In:Ga_2_O_3_ target with a 10% In content was utilized to deposit the In:Ga_2_O_3_ thin films, and it was positioned 6 cm away from the substrate. The KrF excimer laser emitted deep ultraviolet light with a wavelength of 248 nm, a frequency of 2 Hz, and a fluence of 300 mJ. This light was focused by the optical system and then acted on the In:Ga_2_O_3_ target. The substrate temperature was maintained at 650 °C, and the deposition time was 60 min. Prior to growth, the background vacuum of the depositing chamber was pumped to 10^−5^ Pa and then fed into the pure oxygen gas in the chamber. By controlling the flow rate of oxygen, the pressure in the depositing chamber with 0.1 Pa, 1 Pa, 2 Pa, 5 Pa, and 10 Pa was obtained for depositing different In:Ga_2_O_3_ layers. Under the same deposition conditions, the Ga_2_O_3_/p-GaN heterojunction without In doping was also prepared with 10 Pa oxygen pressure as reference samples for this study. Using the thermal evaporation method, two pairs of 80 nm-thick interdigitated Au electrodes were prepared on the In:Ga_2_O_3_ and p-GaN films via a mask plate to construct the quasi-vertical p–n junction device based on the In:Ga_2_O_3_/p-GaN heterojunction. The length and width of a single-finger interdigitated electrode are 500 μm and 10 μm, respectively. The photosensitive area is 0.09 cm^2^. The schematic diagram of the photodetector is shown in Figure 1a. The measurement of heterojunction devices is performed by connecting one interdigitated electrode of the In:Ga_2_O_3_ layer as the cathode and one interdigitated electrode of the GaN layer as the anode. In this case, the voltage direction is defined as positive voltage, and the generated current is positive current. The opposite corresponds to negative voltage and negative current.

To investigate the phase structures, X-ray diffraction (XRD) analyses were conducted using a Rigaku SmartLab SE9KW X-ray diffractometer equipped with a 9 kW rotating anode X-ray source and Cu K_α_ lines as the X-ray source. The transmittance and absorption of the sample were tested with a Lambda-950 UV/Vis/NIR spectrophotometer of PerkinElmer Company. The surface morphology of the sample was analyzed using a HITACHI SU-70 field-emission scanning electron microscope (SEM). The surface roughness was examined with a Bruker Dimension ICON atomic force microscope (AFM). Spectral responsivity and current–voltage (I–V) characteristics were measured using a Zolix Solar Cell Scan100 spectral response testing system.

## 3. Results and Discussion

### 3.1. Preparation and Characterization of In:Ga_2_O_3_/p-GaN Heterojunction Solar-Blind Ultraviolet Detector

Figure 1b displays the XRD patterns of the In:Ga_2_O_3_/p-GaN sample prepared at an oxygen pressure of 5 Pa. The diffraction peaks mainly originate from the GaN layer. No obvious diffraction peak related to Ga_2_O_3_ is observed. A pulsed laser has high energy density to rapidly elevate the target material surface temperature, leading to its evaporation. Numerous reactive particles are generated within the chamber and subsequently diffuse towards the substrate. During their diffusion towards the substrate, the reactive particles continuously collide with the oxygen atoms present in the chamber. During this process, oxygen atoms participate in reactions to form stable chemical bonds, while excessive collisions result in substantial energy loss among the reactive particles. Higher oxygen pressure increases collision probabilities between reactive particles and oxygen atoms, leading to a reduction in migration energy. When those particles reach the substrate, they do not possess enough migration energy to reach the appropriate crystallization positions, which impedes grain growth and consequently leads to a deterioration in the crystalline quality of thin films. The film, ultimately, is composed of numerous microcrystals and nanocrystals, which render the detection of crystalline diffraction peaks difficult. The inset in Figure 1b displays the SEM images of the In:Ga_2_O_3_/p-GaN sample. It can be observed that the film consists of microcrystalline and nanocrystalline clusters and features a flat surface, as detailed in Figure S2d (Supplementary Materials). X-ray photoelectron spectroscopy (XPS) was employed to analyze the detailed chemical composition of the In:Ga_2_O_3_ films. Figure 1c shows the O 1s deconvoluted spectrum, while the inset in Figure 1c presents the In 3d spectrum. The core-level spectra in Figure 1c reveal three distinct oxygen valence states. O_I,_ O_II,_ and O_III_ correspond to the oxygen atoms occupying the lattice, oxygen vacancies, and free oxygen atoms in the crystal, respectively [38,39]. The binding energies of O_I,_ O_II,_ and O_III_ are 529.4 eV, 530.8 eV, and 532.1 eV, respectively. The proportions of O_I,_ O_II,_ and O_III_ in the In:Ga_2_O_3_ films are 7.3%, 49.1%, and 43.6%, respectively. Compared with the XPS results of the Ga_2_O_3_ film (shown in Figure S4a), In doping leads to an increase in the proportion of O_II_ and O_III_, indicating that In doping can increase the concentration of oxygen vacancies. Because oxygen vacancies can provide electrons [40], In doping can improve the conductivity of the film. The inset of Figure 1c shows that the binding energy of In 3d is 445.5 eV. It is reported that the binding energy of the In-O bond in In_2_O_3_ is 444.6 eV, which is very close to the result in Figure 1c. It is confirmed that In atoms mainly combine with oxygen atoms to form In-O bonds or In-O-Ga bonds [41], which increases the carrier concentration in the film and improves the conductivity. Figure 1d illustrates the spectral responsivity of the In:Ga_2_O_3_/p-GaN heterojunction device under various reverse bias conditions. A 254 nm ultraviolet light source with a power of 128 μW/cm^2^ was used to irradiate the surface of the In:Ga_2_O_3_. From the response spectra, it is evident that the device exhibits two corresponding peaks in the solar blind UV band (~250 nm) and the near UV band (~345 nm), which come from the In:Ga_2_O_3_ layer and p-GaN substrates, respectively. As the reverse bias increases, the intensity of the response peak exhibits a rapid rise. Specifically, at a reverse bias of −2.5 V, the responsivity peaks at 250 nm and 345 nm are measured to be 0.19 A/W and 0.036 A/W, respectively. The response peaks originating from In:Ga_2_O_3_ are dominant in the response spectra. The device also performs the self-powered effect, which is attributed to the built-in electric field formed at the In:Ga_2_O_3_/p-GaN heterojunction. Under zero bias, the self-powered response peaks located at 250 nm and 345 nm are 3.5 × 10^−4^ A/W and 1.1 × 10^−3^ A/W, which indicates that the GaN layer dominates the spectral response. These results demonstrate that the spectral responsivity of heterojunctions can be significantly enhanced, and the modulation of the response peak position can be achieved by applying a small reverse bias. The detectivity (D*) can be calculated using the calculation $D^{*}=R{\left(S/2eI_{dark}\right)}^{1/2}$, where e is the elemental charge, S is the photosensitive area, and I_dark_ is the dark current [42]. A maximum value of 5.1 × 10^10^ Jones is obtained under 254 nm light illumination, with a power of 128 μW/cm^2^ at −2.5 V bias. The noise-equivalent power (NEP) is calculated to $5.9\times 10^{-12}W/{Hz}^{1/2}$, using the calculation $NEP=S^{1/2}/D^{*}$. The external quantum efficiency (EQE) is calculated to 94.6%, using the calculation $EQE=\frac{hcR}{q\lambda }\times 100$. The UV/visible rejection ratio is calculated to be 8.26, using the responsivity (R) at 250 nm and 400 nm from Figure 1d. Figure 1e presents the I–V characteristic curves of an In:Ga_2_O_3_/p-GaN heterojunction device prepared with an oxygen pressure of 5 Pa in the dark and under ultraviolet light illumination. The photocurrent was carried out under the illumination of a 254 nm ultraviolet light source with a power of 128 μW/cm^2^. The I–V curve exhibits distinct rectification characteristics in the dark, indicating that the In:Ga_2_O_3_/p-GaN junction functions as a well-defined diode. It shows a significantly enhanced ratio of photo current to dark current at zero and small reverse bias. The photocurrent reaches 0.2 µA at zero bias, which is due to the generation and collection of photo-generated carriers. In solar cells, the photo-current under zero bias is called the short-circuit current, which is an important parameter for solar cells. The In:Ga_2_O_3_/p-GaN heterojunction shows a large short circuit (about 0.2 µA) under UV illumination, exhibiting the advantage of self-powered characteristics. Meanwhile, the ratio (I_light_/I_dark_) of photo current and dark current can reach 3.6 × 10^3^, as a small reverse bias is applied to the device. These results indicate that the In:Ga_2_O_3_/p-GaN junction device can be self-powered or operate under a small reverse bias. Figure 1f shows the time-dependent photoresponse by intermittently turning on and off the 254 nm UV illumination with an intensity of 128 μW/cm^2^ under an applied bias of −2.5 V. It is noted that the time-dependent photoresponse shows good reproducibility and stability. The rise time and decay time are estimated by the time interval corresponding to the current of 10% to 90% [39]. According to the results shown in Figure 1f, the rise time and fall time are estimated to be 0.79 S and 7.40 S, respectively.

### 3.2. The Effect of Oxygen Pressure on Spectral Response Characteristics of In:Ga_2_O_3_/p-GaN Heterojunction

Although the In:Ga_2_O_3_/p-GaN heterojunction device has shown good performance, further investigation into the impact of oxygen pressure on the properties of In:Ga_2_O_3_ thin films is essential. This is because oxygen atoms play a dual role as both a reactive species in the deposition process and a determinant of the film properties, which can subsequently affect the performance of heterojunction detectors. To investigate the effect of oxygen pressure on the performance of an In:Ga_2_O_3_/p-GaN heterojunction device, a series of In:Ga_2_O_3_/p-GaN heterojunction devices is tested under the oxygen pressures of 0.1 Pa, 1 Pa, 2 Pa, 5 Pa, and 10 Pa, respectively. The In:Ga_2_O_3_ films have significant visible-light transmittance and UV absorption characteristics, as shown in Figure S1. The bandgaps of the In:Ga_2_O_3_ film are estimated to be 4.5 eV to 4.8 eV for different deposition oxygen pressures (see Figure S1d). Table 1 shows the resistivity and thickness of In:Ga_2_O_3_ films with different deposition oxygen pressures. The resistivity of In:Ga_2_O_3_ film increases quickly with the increase in oxygen pressure. The reason is that the increase in oxygen pressure leads to a higher collision probability, which can degrade the crystal quality and result in a reduction of In content, as shown in Figure S2. The thickness of In:Ga_2_O_3_ films decreases with an increase in the oxygen pressure, indicating that high collision probability is not conducive to the formation of thin films.

**Figure 2:**
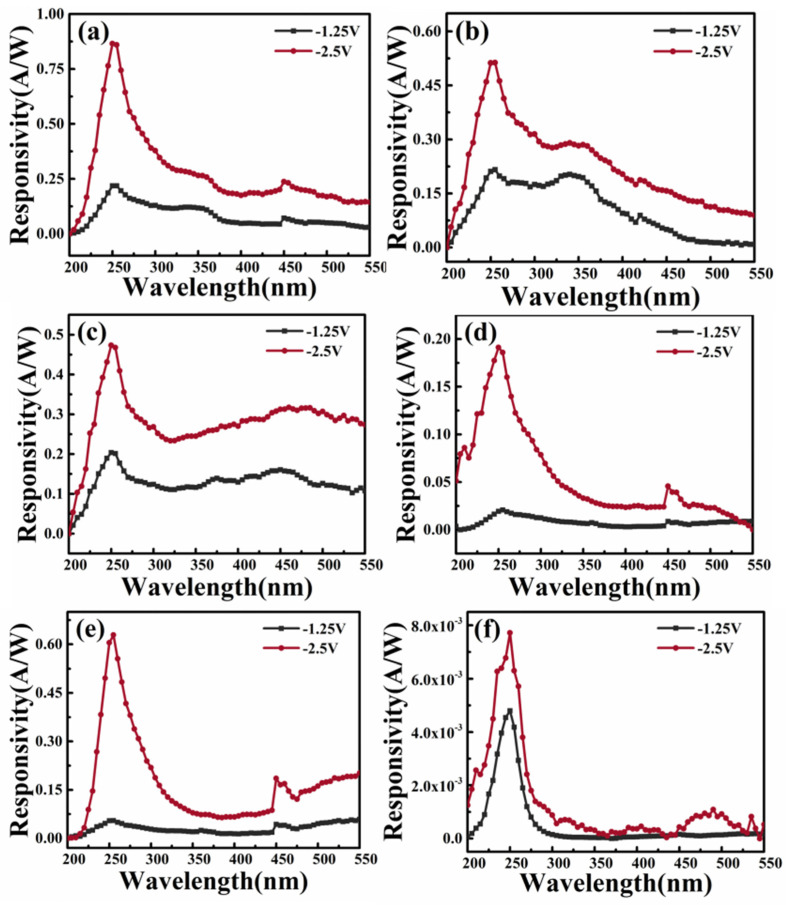
The spectral responsivity of In:Ga_2_O_3_/p-GaN heterojunctions under −2.5 V and −1.25 V reverse bias with different deposition oxygen pressures: (**a**) 0.1 Pa; (**b**) 1 Pa; (**c**) 2 Pa; (**d**) 5 Pa; (**e**) 10 Pa; (**f**) 10 Pa (In% = 0).

Figure 2 shows the spectral responsivity of In:Ga_2_O_3_/p-GaN heterojunctions prepared with different deposition oxygen pressure under −2.5 V and −1.25 V bias. It is found that the In:Ga_2_O_3_/p-GaN heterojunction detectors have a wide response peak in the range of 240~400 nm. It can be observed from the response spectra that the device contains two corresponding peaks in the solar-blind UV band and the near UV band, which come from the In:Ga_2_O_3_ and p-GaN substrates, respectively. It can be observed from Figure 2 that the responsivity of all In:Ga_2_O_3_/p-GaN heterojunctions is significantly improved as a small reverse bias is applied to the structure. The reverse bias leads to an increase in the thickness of the spatial-charge region on both sides of the junction. Because the photo-generated carriers are mainly separated in the spatial-charge region, the increase in the reverse bias can increase the responsivity. At the same time, the position of the predominant light response peak also changed significantly. At low oxygen pressure, the intensity of the response peak located at 250 nm increases significantly and gradually becomes the predominant peak in the response spectra as the reverse bias increases. At high oxygen pressure, the spectral response curve of the heterojunction detector has a relatively narrow spectral range, and its predominant peak position is located in the solar-blind UV region. The change in responsivity is related to the crystalline quality and In content of the In:Ga_2_O_3_ layer, which is related to the oxygen pressure during the deposition process. With the increase in the oxygen pressure, the crystalline quality of In:Ga_2_O_3_ films decreases due to the reduction of reactive particle migration energy, which is not conducive to In doping and leads to an increase in the optical bandgap of In:Ga_2_O_3_ thin films. The detailed results are presented in Figure S1 of the Supporting Information. The SEM and AFM images of the In:Ga_2_O_3_ films indicate that the crystal grains become smaller and the surface of the film becomes flat, as the oxygen pressure increases, as seen in Figures S2a–d and S3. As estimated from the EDS elemental spectrum shown in the inset of Figure S2, the In content in the film decreases as the oxygen pressure increases (see Figure S2f), resulting in a decrease in the conductivity and carrier concentration. Low electron concentration and high resistivity are not conducive to the transmission and effective collection of photo-generated carriers. Therefore, the responsivity of In:Ga_2_O_3_/p-GaN heterojunction devices decreases with the increase in the oxygen pressure, as shown in Figure 2a–d. The incorporation of In will also cause the generation of defect states, such as oxygen vacancies. The oxygen vacancy and the interface states located at the grain boundaries should capture carriers to hinder their recombination, which increases the photo-current and responsivity [37,43,44]. It is noted that the responsivity of the device prepared with an oxygen pressure of 10 Pa is relatively large. The main reason is that the film is composed of microcrystals and nanocrystals (see Figures S2e and S3e), which may lead to more interface states that can produce internal gain [45,46]. As a comparison, the heterojunction device without In doping has better solar-blind spectral selection characteristics, but the responsivity is much lower than that of the In:Ga_2_O_3_/p-GaN heterojunction devices. The responsivities of the In:Ga_2_O_3_/p-GaN and Ga_2_O_3_/p-GaN heterojunction are 0.63 A/W and 7.7 × 10^−3^ A/W, respectively, as a reverse bias of 2.5 V is applied on the device. The results indicate that introducing In atoms into the Ga_2_O_3_ films can greatly improve the device’s performance. Under the same growth conditions, In:Ga_2_O_3_ films have a higher carrier concentration and better electrical properties, resulting in better responsivity.

**Figure 3:**
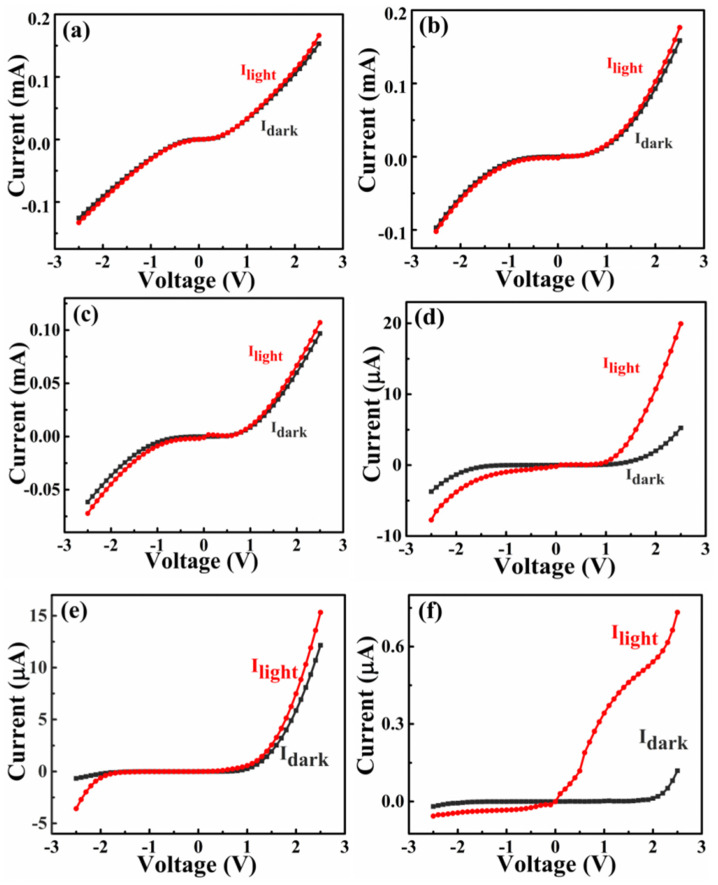
The I–V curves of In:Ga_2_O_3_/p-GaN heterojunctions with different deposition oxygen pressures: (**a**) 0.1 Pa; (**b**) 1 Pa; (**c**) 2 Pa; (**d**) 5 Pa; (**e**) 10 Pa; (**f**) 10 Pa (In% = 0).

Figure 3 illustrates the I–V characteristics of In:Ga_2_O_3_/p-GaN heterojunctions fabricated under varying oxygen pressures during deposition. The photo-current measurement was conducted under the irradiation of a 254 nm ultraviolet light source with a power density of 128 μW/cm^2^. At low oxygen pressure (0.1~2 Pa), the In:Ga_2_O_3_ thin films exhibit low resistance due to the higher In content, as shown in Figure S2f. The dark current can reach the order of mA at 2.5 V bias, as shown in Figure 3a–c. A higher dark current makes it difficult to distinguish photo–dark current, resulting in a small ratio of photo-to-dark current under forward bias. Under reverse bias, a significant reverse leakage current occurs due to the thinner spatial-charge region on the In:Ga_2_O_3_ side, resulting in insignificant rectification characteristics. The main reason is that, under low oxygen pressure, the spatial-charge region of In:Ga_2_O_3_ is very thin due to the high In composition, resulting in a tunneling current under reverse bias. The existence of a large leakage current makes the ratio of photo-to-dark current very small. When the oxygen pressure is high (5~10 Pa), the resistance of the In:Ga_2_O_3_ thin film increases, and the carrier concentration decreases, which is attributed to the reduction in In doping concentration and degradation of crystal quality. The photo and dark current can decrease to the order of µA, as shown in Figure 3d,e. The heterojunctions exhibit obvious semiconductor rectification characteristics and show a good p–n junction effect. Due to the cut-off characteristics of the p–n junction under reverse bias, the dark current is relatively low. In this case, the photo-generated carriers produced by light illumination can form a large photo-current gain. As a result, the devices exhibit a high photo-to-dark current ratio up to three orders of magnitude under a small negative bias condition. In addition, it can be seen from Figure 3d–f that a pronounced separation between the photo and dark currents is observed under forward bias. This is due to the low dark current caused by the high resistance of the In:Ga_2_O_3_ layer. It is worth noting that, although the photo-current has increased, the photo-to-dark current ratio only reaches one order of magnitude, which is much smaller than the ratio under reverse bias. These results indicate that the device can achieve a higher signal-to-noise ratio under a small reverse bias, making the device suitable for operation under a small reverse bias.

**Figure 4:**
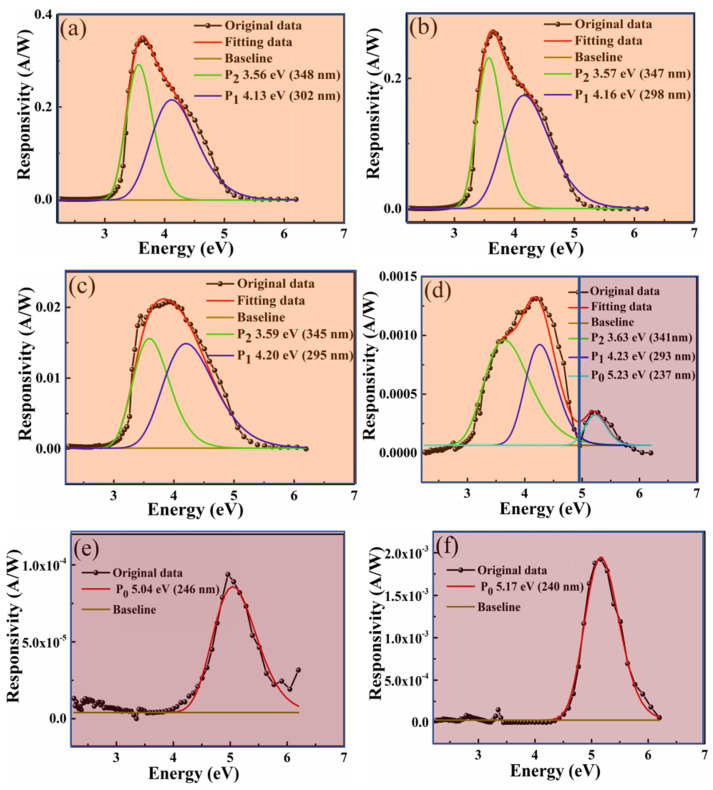
The spectral responsivity of In:Ga_2_O_3_/p-GaN heterojunctions with different deposition oxygen pressures under zero bias: (**a**) 0.1 Pa; (**b**) 1 Pa; (**c**) 2 Pa; (**d**) 5 Pa; (**e**) 10 Pa; (**f**) 10 Pa (In% = 0).

The ability to detect ultraviolet light without an external electric field represents a significant advantage for heterojunction devices. The In:Ga_2_O_3_/p-GaN heterojunction devices exhibit a pronounced self-powered effect. Figure 4 illustrates the spectral responsivity of In:Ga_2_O_3_/p-GaN heterojunction devices fabricated under different deposition oxygen pressures at zero bias. The self-powered response effect is clearly observed from the diagram. It is found that the In:Ga_2_O_3_/p-GaN heterojunction detectors have a wide self-powered response peak in the range of 240~400 nm (3.10 eV~5.17 eV). From the response spectra, it can be observed that the device has two corresponding peaks in the solar-blind UV band and the near UV band, which originate from the In:Ga_2_O_3_ and p-GaN substrates, respectively. In order to study the self-powered response effect, Gaussian multi-peak fitting was performed on the spectrum. For multi-peak fitting rigorously [47], Figure 4 shows the relationship between the spectral responsivity and the photon energy. It is found that the response peaks related to p-GaN intrinsic absorption (P_2_, near 3.59 eV, or 345 nm), defect state (P_1_, near 4.13 eV, or 300 nm), and In:Ga_2_O_3_ intrinsic absorption (P_0_, near 4.96 eV, or 250 nm) are observed on the response spectrum. At low oxygen pressure, the intensity of the P_2_ response peak is relatively strongest, indicating that the photo-generated carriers are mainly formed in the p-GaN layer. The intensity of the P_0_ peak in the response spectrum is relatively weak, as shown in Figure 4a–c. However, with the increase in oxygen pressure, the P_0_ response peak related to the In:Ga_2_O_3_ intrinsic absorption gradually intensifies relative to P_1_ and P_2_. When the oxygen pressure increases to 10 Pa, the spectral response almost entirely comes from the In:Ga_2_O_3_ layer, as shown in Figure 4d,e. The results indicated that the spectral response can be tuned from 3.59 eV (345 nm) to 4.96 eV (250 nm) by altering the oxygen pressure during pulsed laser deposition. As mentioned above, oxygen pressure has a significant effect on the In content and the electrically conducting properties of the In:Ga_2_O_3_ films. The variation in carrier concentration within the films, induced by different In contents, is a key factor contributing to the shift in the spectral response peak. Through the control of growth parameters for the In:Ga_2_O_3_/p-GaN heterojunction, the modulation of the detector’s light response wavelength is achieved. The appearance of the P_1_ peak in the response spectrum suggests that there are more defect states related to oxygen vacancy (V_O_) in the In:Ga_2_O_3_ layer as a result of the In-doping effect and oxygen pressure. As shown in Figure S4, XPS results confirm that the concentration of oxygen vacancy increases with oxygen pressure and In doping. The relative intensity of the P_1_ peak (relative to the P_0_ and P_2_ peaks) in the response spectrum increases with the oxygen pressure, as shown in Figure 4a–d. As the oxygen pressure further increases, the defect state degenerates to form a continuous state, which is located at the bottom of the conduction band. P_0_ and P_1_ peaks are combined into a response peak with a high full width at half maximum (FWHM), as shown in Figure 4e. From Figure 4, it is found that the responsivity of the In:Ga_2_O_3_/p-GaN heterojunctions decreases with the increase in oxygen pressure. The maxima of the self-powered responsivity are approximately 0.35 A/W, 0.28 A/W, 0.021 A/W, 1.3 mA/W, and 0.09 mA/W, corresponding to oxygen pressures of 0.1 Pa, 1 Pa, 2 Pa, 5 Pa, and 10 Pa, respectively. This result is attributed to the decrease in photo-current, which is due to the reduction in conductivity as the deposited oxygen pressure increases. The Ga_2_O_3_/p-GaN heterojunction was also prepared under an oxygen pressure of 10 Pa with the same growth parameters for a comparative study. From Figure 4f, it can be seen that the self-powered responsivity of Ga_2_O_3_/p-GaN heterojunction is 1.9 mA/W, which is higher than that of the In:Ga_2_O_3_/p-GaN heterojunction prepared under the same growth parameters. The full FWHM values are lower than those of the In:Ga_2_O_3_/p-GaN heterojunctions. These results indicate that the Ga_2_O_3_ thin film has a relatively higher crystal quality due to the absence of In atoms, leading to better self-powered solar-blind ultraviolet detection performance. In contrast, the In:Ga_2_O_3_ thin film prepared under the same growth parameters primarily consists of microcrystalline and nanocrystalline clusters, exhibiting more defect states. It has a greater dark current while achieving a certain optical gain, so the self-powered responsivity is relatively lower [33,34]. In addition, it is noted that, under high oxygen pressure, the self-powered response peak comprises a forward response current in the solar-blind ultraviolet region and a reverse response current in the near-ultraviolet region, indicating that there exists complex photoresponse mechanisms at the heterojunction. It is necessary to further study the photoresponse mechanism of In:Ga_2_O_3_/p-GaN heterojunctions.

### 3.3. Study on the Photoresponse Mechanism of In:Ga_2_O_3_/GaN Heterojunction Detectors

**Figure 5:**
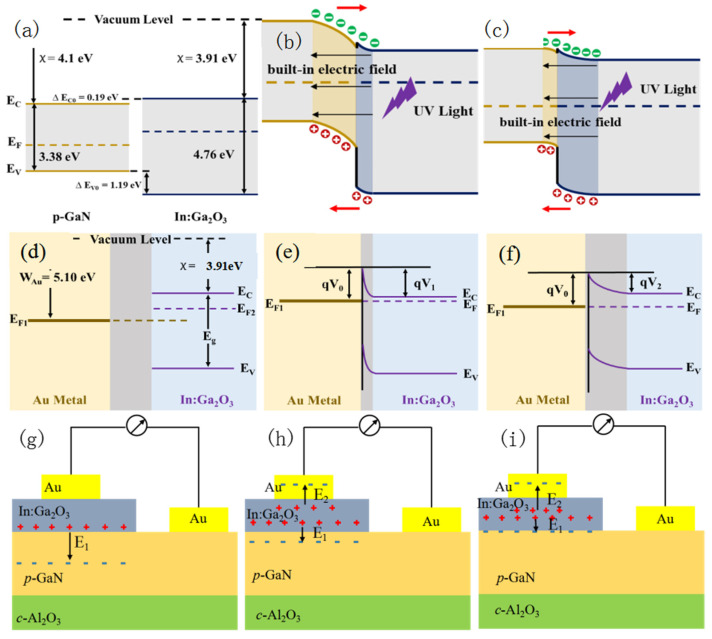
Schematic energy band diagrams of In:Ga_2_O_3_/p-GaN heterojunction: (**a**) before contact; (**b**) upon contacting p-GaN with In:Ga_2_O_3_ prepared under low oxygen pressure; (**c**) upon contacting p-GaN with In:Ga_2_O_3_ prepared under high oxygen pressure. Schematic energy band diagrams of In:Ga_2_O_3_/Au: (**d**) before contact; (**e**) upon contacting Au with In:Ga_2_O_3_ prepared under low oxygen pressure; (**f**) upon contacting Au with In:Ga_2_O_3_ prepared under high oxygen pressure. Self-powered schematics of In:Ga_2_O_3_/p-GaN heterojunction prepared under different oxygen pressures: (**g**) low oxygen pressure; (**h**) moderate oxygen pressure; (**i**) high oxygen pressure.

In order to understand the self-powered effect and the tunable photoresponse spectra effect in the In:Ga_2_O_3_/p-GaN heterojunctions, the schematic diagram of the carriers’ generation and separation is shown in Figure 5. As reported by others, the bandgaps of GaN, Ga_2_O_3_, and In_2_O_3_ are 3.38 eV, 4.9 eV, and 3.13 eV, respectively, and the electron affinities are 4.1 eV, 4.0 eV, and 2.9 eV, respectively [28]. Research indicates that the electronic bandgap of (In_x_Ga_1−x_)_2_O_3_ exhibits a linear relationship with the indium content [29]. By using the atomic percentages of In and Ga at an oxygen pressure of 10 Pa in Figure S2f, the bandgap and electron potential of In:Ga_2_O_3_ are calculated to be 4.76 eV and 3.91 eV, respectively. Then, the conduction band offset is 0.19 eV, and the valence band offset is 1.19 eV, indicating that the type of band alignment is type-I [48]. Figure 5a shows the energy-band diagram of the p-type GaN and n-type In:Ga_2_O_3_ before contacting to form a p–n junction. When they are in contact, some electrons diffuse from the In:Ga_2_O_3_ layer to the p-GaN side, while holes diffuse from the p-GaN layer to the In:Ga_2_O_3_ side. Ultimately, positively and negatively charged ions will remain in the n-type and p-type regions, respectively, thereby forming a spatial-charge region that generates a built-in electric field. The built-in electric field is directed from the n-type In:Ga_2_O_3_ side to the p-GaN side. Upon exposure to UV light, a significant number of photo-generated electron–hole pairs are created within the diode region. These electrons and holes are quickly separated by the built-in electric field, resulting in the generation of a photo-current [49]. As mentioned above, at low oxygen pressure, the In:Ga_2_O_3_ thin films exhibit low resistance owing to their higher In content. It reveals that the electron concentration is high. Hence, the spatial-charge region width on the In:Ga_2_O_3_ side is smaller than that on the p-GaN side, as shown in Figure 5b. This indicates that the photo-generated carriers are primarily separated and collected in the GaN layer, leading to the self-powered spectral response of the device being dominated by GaN-related P_2_ peaks. However, the In:Ga_2_O_3_ thin films prepared with high oxygen pressure exhibit a high resistance and low electron concentration, which results in the spatial-charge region width on the In:Ga_2_O_3_ side being much larger than that on the p-GaN side, as shown in Figure 5c. This results in the photo-generated carriers being mainly separated in the In:Ga_2_O_3_ layer, and thus, the self-powered spectral response of the device is predominantly determined by the P_0_ peak related to In:Ga_2_O_3_. When a reverse bias is applied to the devices, the thickness of the spatial-charge region on both sides of the junction will increase. For the device prepared with low oxygen pressure, the increase in the width of the spatial-charge region in the In:Ga_2_O_3_ layer is larger than that in the GaN layer due to the effect of minority carrier extraction. Therefore, as the reverse bias increases from 0 to −2.5 V, the width of the spatial-charge region in the In:Ga_2_O_3_ thin layer changes from being less than to being greater than that in the GaN layer. This can be used to explain that the predominant peak position of the light response peak also gradually changes from P_2_ to P_0_ with the increase in reverse bias, as shown in Figure 2.

Notably, under zero bias, the device exhibits a positive response current at the P_0_ response peak. As depicted in Figure 4, the positive response current region is marked in purple, while the negative response current region is marked in orange. The presence of a positive response current at the P_0_ response peak indicates that the electric field direction for separating photo-generated carriers is positive, directing from the p-type region to the n-type region, as mentioned above. This electric field direction is opposite to the built-in electric field direction, indicating that there is another mechanism to excite the electric field. Analysis indicates that the Schottky junction effect resulting from the contact between the metal and the semiconductor is the mechanism for the formation of the positive electric field. The I–V measurement results shown in Figure S5 confirm that there is a Schottky contact between the metal Au and the In:Ga_2_O_3_ film deposition under high oxygen pressure (5~10 Pa). As shown in the schematic diagram of Figure 5d, the work function of metal Au is about 5.1 eV, larger than that of In:Ga_2_O_3_. When Au comes into contact with In:Ga_2_O_3_ prepared with a low oxygen pressure, the higher electron concentration due to the higher In content in the In:Ga_2_O_3_ layer makes the barrier region very thin. As a result, carriers can pass through the barrier region via the tunneling effect, forming a large tunneling current, as shown in Figure 5e. In this case, the contact between the Au metal and In:Ga_2_O_3_ is an ohmic contact, and the contact barrier can be ignored. However, when Au contacts with In:Ga_2_O_3_ prepared by high oxygen pressure, the electron concentration is low due to the low In content in the In:Ga_2_O_3_ layer, which makes the barrier region thicker and forms an obvious electric field from the In:Ga_2_O_3_ layer to the Au metal, as shown in Figure 5f. Hence, at low oxygen pressure, there is only a built-in electric field (E_1_) from In:Ga_2_O_3_/p-GaN heterojunctions, as shown in Figure 5g. The separated photo-generated carriers on the GaN side form a negative response current driven by the built-in electric field, which corresponds to the spectra response shown in Figure 4a–c. As the oxygen pressure increases, a Schottky contact forms between the In:Ga_2_O_3_ film and the Au electrode, accompanied by a Schottky barrier, which generates another built-in electric field (E_2_) from the In:Ga_2_O_3_ layer to the Au electrode. As shown in Figure 5h,i, E_1_ and E_2_ are oriented in opposite directions. When the oxygen pressure is moderate, the spatial-charge region is distributed in both the GaN layer and the In:Ga_2_O_3_ layer. The photo-generated carriers generated on the GaN layer and the In:Ga_2_O_3_ layer are affected by both E_1_ and E_2_. At the same time, as the oxygen pressure increases, the electric field E_2_ formed by the Schottky barrier gradually becomes stronger, and the photo-generated carriers from the In:Ga_2_O_3_ side are mainly affected by E_2_. The positive response current gradually increases. The photo-generated carriers from the GaN side are primarily influenced by the built-in electric field E_1_, resulting in a gradual decrease in the negative response current. This corresponds to the spectral response shown in Figure 4d. When the oxygen pressure further increases to 10 Pa, the resistance of In:Ga_2_O_3_ becomes larger. Due to the Schottky contact with the Au electrode, the spatial-charge region formed on the surface of the In:Ga_2_O_3_ film becomes wider. On the other hand, the spatial-charge region between the In:Ga_2_O_3_/p-GaN heterojunction has mainly formed on the In:Ga_2_O_3_ side. Although both of them generate a solar-blind ultraviolet light response from the In:Ga_2_O_3_ layer, the current direction is opposite. It is noted that the photoresponse current is positive at this time, indicating that the main photoresponse comes from the Schottky barrier effect. This is primarily because ultraviolet light irradiates the surface of the In:Ga_2_O_3_ layer, and the absorption intensity gradually decreases from the In:Ga_2_O_3_ surface to the In:Ga_2_O_3_/p-GaN junction interface, resulting in the optical gain caused by E_2_ being stronger than that of E_1_. The photo-generated carriers from intrinsic absorption are dominated by the positive response current generated by the action of E_2_ on the In:Ga_2_O_3_ surface, as shown in Figure 5i. This indicates that the oxygen pressure can significantly influence the distribution of the spatial-charge region and built-in electric field in the structure, thereby affecting the spectral response characteristics of the In:Ga_2_O_3_/p-GaN heterojunction devices.

**Figure 6:**
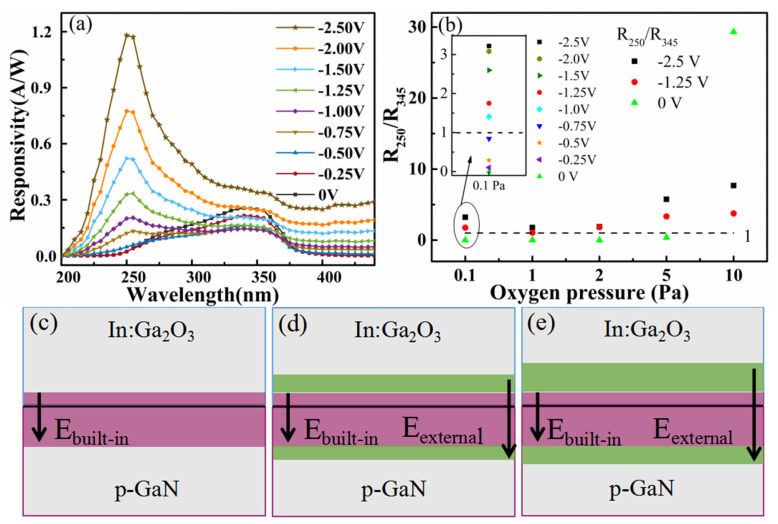
(**a**) The spectral responsivity of In:Ga_2_O_3_/p-GaN heterojunction with 0.1 Pa oxygen pressure under different negative bias; (**b**) the ratio of R_250_ and R_345_ with different oxygen pressure; the schematic diagram of the spatial-charge region and built-in electric field under different reverse bias voltages: (**c**) zero bias; (**d**) moderate reverse bias; (**e**) high reverse bias.

The effect of reverse bias on the responsivity was investigated by applying a reverse bias to the devices. As shown in Figure 2, the responsivity of all In:Ga_2_O_3_/p-GaN heterojunctions is significantly improved when a small reverse bias is performed on the structure. At the same time, the predominant peak position of the light response peak also changed significantly. In order to investigate the effect of reverse bias on the spectral response characteristics of the In:Ga_2_O_3_/p-GaN heterojunction devices, take the sample prepared with 0.1 Pa oxygen pressure as a sample. The effect of reverse bias on the responsivity was studied in detail by applying a series of reverse biases with smaller intervals on the sample. Figure 6a clearly shows the relationship between the intensity of the response peak and the reverse bias. The position of the dominant response peak gradually changes from P_2_ to P_0_, indicating that the photo-generated carriers change from mainly generated in the GaN layer to mainly generated in the In:Ga_2_O_3_ layer. Analysis suggests that the alterations in the spatial-charge region and the built-in electric field caused by reverse bias lead to a change in the response peak intensity and position. As shown in Figure 6c–e, it is a schematic diagram illustrating the change process of the spatial-charge region under reverse bias. The purple area in the figure is the spatial-charge region under zero bias, and the green area is the spatial-charge region under the influence of reverse bias. When the reverse bias is zero, the spatial-charge region is narrow and mainly formed on the GaN side, resulting in the separation of photo-generated carriers on the GaN side. The spectral response wavelength is located in the near ultraviolet region (about 345 nm). At this time, the responsivity in the near ultraviolet region is the largest, which is 0.26 A/W. As the reverse bias increases, the response peak in the near ultraviolet region gradually diminishes, while that in the solar blind ultraviolet region gradually rises. When the bias increases to −0.75 V, the reverse bias causes the spatial-charge region to broaden, making the spatial-charge regions on both sides of the junction equivalent. The peak intensity of the response in the solar blind ultraviolet region is comparable to that in the near-ultraviolet region. When the reverse bias continues to increase to −2.5 V, the spatial-charge region on the In:Ga_2_O_3_ side becomes larger than that on the GaN side. The response is gradually dominated by the solar blind ultraviolet band (near 250 nm). The responsivity of the photodetector is 1.18 A/W. The ratio of responsivity (R) at 250 nm and 345 nm (R_250_/R_345_) is utilized to characterize the transition of the response peak and its correlation with the reverse bias. When the value of R_250_/R_345_ is less than one, it indicates that the spectral response is predominantly governed by the GaN layer. Conversely, the spectral response is predominantly governed by the In:Ga_2_O_3_ layer. Figure 6b illustrates the relationship between R_250_/R_345_ and both the oxygen pressure and the reverse bias applied to the device. The figure clearly shows that, under zero bias, the R value gradually increases from close to 0 to 29.3 when the oxygen pressure is increased from 0.1 Pa to 10 Pa. Under low oxygen pressure, increasing the reverse bias can also change the R value from less than one to more than one. This demonstrates that the response spectra of the device can be modulated by adjusting the oxygen pressure during deposition and altering the negative bias applied to it. The In:Ga_2_O_3_/p-GaN heterojunctions have demonstrated the tunable capability to be detected in the blind region and near ultraviolet region, displaying the advantageous properties for solar-blind ultraviolet and near-ultraviolet detection applications.

## 4. Conclusions

In this paper, we have prepared a series of PDs based on an In:Ga_2_O_3_/p-GaN heterojunction using the PLD method under different oxygen deposition pressures. Experimental results show that oxygen pressure has a significant effect on the crystallinity and optical and conductivity properties of In:Ga_2_O_3_ films. As the oxygen pressure increases, the carrier concentration and conductivity of the In:Ga_2_O_3_ films decrease because of the reduction in In content and the degradation of crystal quality. The fabricated devices exhibit a wide spectral response across the near ultraviolet region to the solar-blind ultraviolet region and have excellent light detection performance. In the In:Ga_2_O_3_/p-GaN heterojunction detector prepared with an oxygen pressure of 5 Pa, the responsivities of the response peaks located at 250 nm and 345 nm are 0.19 A/W and 0.036 A/W at −2.5 V, respectively. Under low oxygen pressure conditions, the In:Ga_2_O_3_/p-GaN heterojunction PDs under zero bias exhibit a broadband self-powered response in the wavelength range of 250–400 nm. Response peaks related to p-GaN intrinsic absorption (near 345 nm), defect state (near 300 nm), and In:Ga_2_O_3_ intrinsic absorption (near 250 nm) are observed on the response spectrum. However, with the increase in oxygen pressure, the response peaks of In:Ga_2_O_3_ and the defect state increase gradually, while the peaks related to GaN decrease gradually. The response characteristics of the devices under different reverse bias voltages were also investigated. The results show that the response peak in the near ultraviolet region gradually decreases, and the response peak in the solar-blind ultraviolet region gradually increases with the increasing reverse bias to −2.5 V. This result shows that the response spectra of the device can be modulated by adjusting the oxygen pressure during the deposition process and changing the reverse bias applied to the device. The tunable photoresponse spectra effect is attributed to the alterations in the spatial-charge region and built-in electric field caused by oxygen pressure and reverse bias applied to the device. The tunable ultraviolet PDs executed in the solar-blind band and near ultraviolet band can be realized in the In:Ga_2_O_3_/p-GaN heterojunctions. This research provides a method for developing tunable photoresponse peak UV PDs for optoelectronic applications.

## Figures and Tables

**Table 1:** The resistivity and thickness of In:Ga_2_O_3_ films with different deposition oxygen pressures.

Oxygen Pressure (Pa)	0.1	1	2	5	10
Resistivity (Ω∙cm)	2.74	28.2	2.3 × 10^3^	8.24 × 10^4^	1.57 × 10^5^
Thickness (nm)	251	244	140	124	94

## Data Availability

The original contributions presented in this study are included in the article/Supplementary Materials. Further inquiries can be directed to the corresponding author(s).

## Supplementary Materials

The following supporting information can be downloaded at: https://www.mdpi.com/article/10.3390/s26041197/s1. Figure S1. (a) XRD patterns of In:Ga_2_O_3_ deposited with different oxygen pressure; (b) transmittance spectra of In:Ga_2_O_3_ thin films; (c) absorption spectra of In:Ga_2_O_3_ thin films. (d) The plots of (αhν)^2^ vs hν of In:Ga_2_O_3_ thin film. The inset is the optical bandgap of In:Ga_2_O_3_ films. Figure S2. The plane SEM and EDS images of In:Ga_2_O_3_ thin films with different deposition oxygen pressures: (a) 0.1 Pa; (b) 1 Pa; (c) 2 Pa; (d) 5 Pa; (e) 10 Pa; (f) the Ga and In atomic percentage of In:Ga_2_O_3_ thin films. Figure S3. The AFM images of In:Ga_2_O_3_ thin films prepared under different deposition oxygen pressures: (a) 0.1 Pa; (b) 1 Pa; (c) 2 Pa; (d) 5 Pa; (e) 10 Pa; (f) surface roughness of In:Ga_2_O_3_ thin films with different deposition oxygen pressures. Figure S4. The XPS spectra of O 1s from Ga_2_O_3_ and In:Ga_2_O_3_ films with different oxygen pressures: (a) Ga_2_O_3_ with deposition oxygen pressure of 1 Pa; (b) In:Ga_2_O_3_ with deposition oxygen pressure of 1 Pa; (c) In:Ga_2_O_3_ with deposition oxygen pressure of 5 Pa. Figure S5. The I–V curves of In:Ga_2_O_3_/p-GaN heterojunctions with different deposition oxygen pressures measured through a pair of interdigitated electrodes on In:Ga_2_O_3_ surface: (a) 0.1 Pa; (b) 1 Pa; (c) 2 Pa; (d) 5 Pa; (e) 10 Pa; (f) 10 Pa (In% = 0).
