# Supplementary material for: Research on Tunable Ultraviolet Detector and Photoresponse Mechanism Based on In:Ga2O3/p-GaN Heterojunction
Source: Sensors (Basel). 2026 Feb 12;26(4):1197. doi: 10.3390/s26041197 (PMC12944459; doi:10.3390/s26041197)
Supplement: Supplementary file 1 [file sensors-26-01197-s001.zip › sensors-4114215-supplementary.pdf]

Supplementary Materials

Although the In:Ga_2O_3/p-GaN heterojunction device has shown good performance, further investigation into the impact of oxygen partial pressure on the properties of In:Ga_2O_3 thin films is essential. This is because oxygen atoms play a dual role as both a reactive species in the deposition process and a determinant of the film properties, which can subsequently affect the performance of heterojunction detectors. To investigate the influence of oxygen pressure on the growth mechanism of In:Ga_2O_3 thin films, we employed the pulsed laser deposition (PLD) technique to deposit these films on c-Al_2O_3 substrates under varying oxygen pressure conditions. The XRD patterns are presented in Figure S1a. At low oxygen pressures (0.1 Pa-1Pa), there are diffraction peaks presented at 2θ of around 18.80°, 38.15° and 58.99°, corresponding to the ($\bar{2}01$), ($\bar{4}02$), and ($\bar{6}03$) lattice planes of monoclinic β-Ga_2O_3. The diffraction peaks at 30.82° related to (400) lattice planes of β-Ga_2O_3 also appear, confirming the formation of β-Ga_2O_3 in the films. The diffraction peak intensity slightly decreases with an increase in the oxygen pressure from 0.1 Pa to 1 Pa, which indicates that the film exhibits good crystallinity under low oxygen pressure. When the oxygen pressure is relatively high (2 to 10 Pa), no obvious diffraction peaks related to Ga_2O_3 are observed. The main reason is that, under high oxygen pressure, the insufficiency of reaction particles' migration energy arises from the increased collision probability, which impedes grain growth and results in a decline in the crystal quality of the film. Figure S1b-d shows the optical properties of In:Ga_2O_3 thin films. The transmittance spectra of In:Ga_2O_3 thin films shown in Figure S1b indicate that the In:Ga_2O_3 films have significant visible light transmittance, and the transmittance reaches 90%. The transmittance in the ultraviolet region drops sharply. As shown in Figure S1c, the In:Ga_2O_3 thin films exhibit sharp absorption edges in the UV region. The inset displays the optical bandgap of In:Ga_2O_3 films, which is evaluated based on the absorption spectra. The optical bandgap of In:Ga_2O_3 films increases with increasing oxygen pressure.

Figure S2a-e shows the SEM images of the In:Ga_2O_3 films. It is noted that the surface particles become denser at the relatively low oxygen pressure (0.1~1Pa). When the oxygen pressure rises from 2 Pa to 5 Pa, the film's flatness improves, and the surface grains become finer. The reason is that increased oxygen pressure leads to a higher collision probability, which can degrade the crystal quality and result in a reduction in grain size. When the oxygen pressure increases to 10 Pa, the grains on the surface of the film agglomerate, and the microcrystalline or nanocrystalline clusters appear as shown in Figure S2. Figure S2f presents the atomic percentages of Ga and In in In:Ga_2O_3 thin films at different deposition oxygen pressures, as estimated from the EDS elemental spectrum. It is evident that both the ratio of In to Ga atoms and the In content in the film decrease as the oxygen pressure increases.

Figure S3a-e shows the surface morphology of the thin film obtained using an atomic force microscope (AFM) with a scanning area

of 10 × 10 μm. The root-mean-square (RMS) surface roughnesses obtained are 14.8 nm, 3.9 nm, 2.81 nm, 0.815 nm, and 2.04 nm, corresponding to 0.1 Pa, 1 Pa, 2 Pa, 5 Pa, and 10 Pa oxygen pressure. The film prepared under high oxygen pressure exhibits lower roughness. The In:Ga_2O_3 films deposited under low oxygen pressure (0.1Pa, 1Pa) exhibit better crystallinity, which increases the roughness. But the film prepared at 1Pa shows grain refinement, resulting in a uniform and smooth surface with roughness reduced to 3.9 nm. As the oxygen pressure increases from 2 Pa to 5 Pa, excessive collisions further reduce the crystallization quality and the grain size of the film. The film becomes flatter and denser. When the oxygen pressure reaches 10 Pa, these small grains are agglomerated to form microcrystalline or nanocrystalline clusters. The films consist of microcrystalline and nanocrystalline grains, exhibiting a relatively smooth surface with consistently low roughness. As shown in Figure S3(f), the In content also showed the same change trend. This implies that the In content is the factor of surface roughness. In general, oxygen pressure has a significant effect on the crystallinity and optical and electrical properties of In:Ga_2O_3 films.

Figure S4 illustrates the XPS spectra of O 1s from Ga_2O_3 and In:Ga_2O_3 films with different oxygen pressures. The core-level spectra in Figure S4 reveal three distinct oxygen valence states. O_{I}, O_{II}, and O_{III} correspond to the oxygen atoms occupying the lattice, oxygen vacancies, and free oxygen atoms in the crystal, respectively. The binding energies of O_{I}, O_{II}, and O_{III} are 529.4 eV, 530.8 eV, and 532.1 eV, respectively. By the Gaussian multi-peak fitting method, the proportions of three different oxygen valence states were studied. The proportion of O_{I}, O_{II}, and O_{III} in Ga_2O_3 films deposited with an oxygen pressure of 1 Pa is 37.2%, 41.2%, and 21.6%, respectively. The proportions of O_{I}, O_{II}, and O_{III} in In:Ga_2O_3 films deposited with an oxygen pressure of 1 Pa are 21.6%, 44.6%, and 33.8%, respectively. The proportions of O_{I}, O_{II}, and O_{III} in In:Ga_2O_3 films deposited with an oxygen pressure of 5 Pa are 7.3%, 49.1%, and 43.6%, respectively. These results indicate that the incorporation of In atoms can reduce the proportion of O_{I} and increase the proportion of O_{II} and O_{III}. In doping can increase the concentration of oxygen vacancies. The oxygen pressure has a great influence on the oxygen vacancy concentration in the film, as shown in Figure S4(b,c). When the oxygen pressure is low, the probability of collision between the reaction particles and the oxygen molecules is low. So, those particles still have a large migration energy, which makes it easier to occupy the lattice position to form O_{I}. As the oxygen pressure increases, the probability of collision increases, resulting in a decrease in the migration energy of the particles. Therefore, oxygen atoms do not easily occupy the lattice position, resulting in many oxygen vacancy defects (O_{II} + O_{III}).

Figure S5 shows the I–V curves of In:Ga_2O_3/p-GaN heterojunction depositions with different deposition oxygen pressures. The current was measured through a pair of interdigitated electrodes on the In:Ga_2O_3 surface. At low oxygen pressure (0.1~2 Pa), the I–V curves show a good linear relationship, indicating that the contact between the electrode and the film is an Ohmic contact. When the oxygen pressure

is high (5~10 Pa), the I–V curve shows a typical nonlinear relationship and junction characteristics. This confirms that there is a Schottky contact between the metal Au and the In:Ga_2O_3 film.

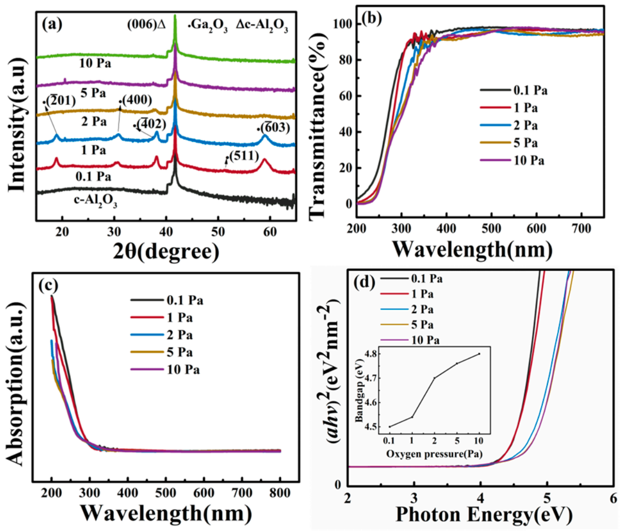

Figure S1. (**a**) XRD patterns of In:Ga_2O_3 deposited with different oxygen pressure; (**b**) transmittance spectra of In:Ga_2O_3 thin films; (**c**) absorption spectra of In:Ga_2O_3 thin films. (**d**) The plots of $(\alpha h\nu)^2$ vs hν of In:Ga_2O_3 thin film. The inset is the optical bandgap of In:Ga_2O_3 films.

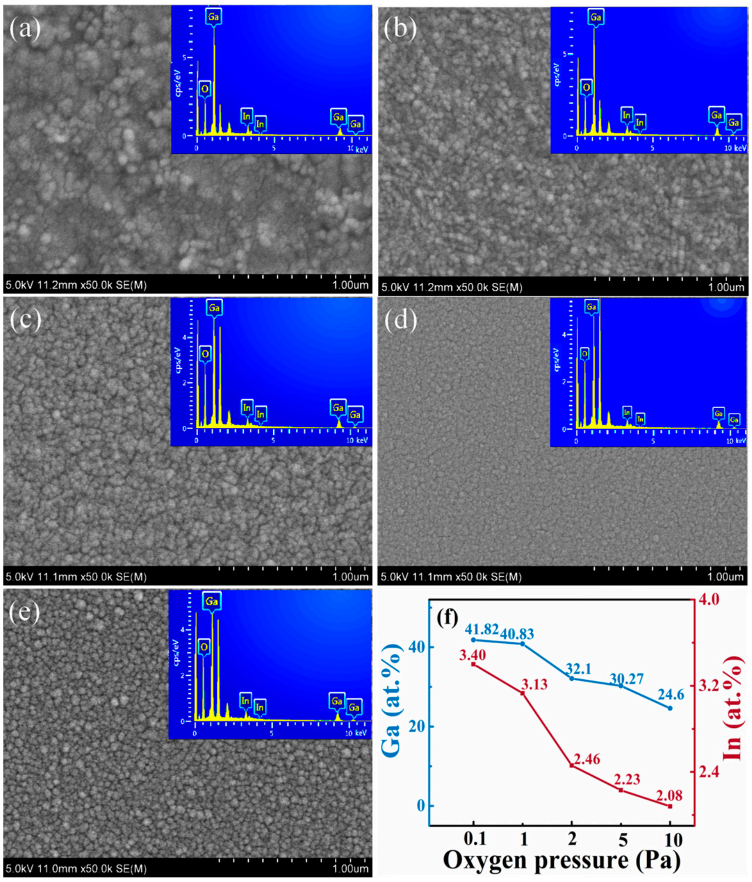

Figure S2. The plane SEM and EDS images of In:Ga_2O_3 thin films with different deposition oxygen pressures: (**a**) 0.1 Pa; (**b**) 1 Pa; (**c**) 2 Pa; (**d**) 5 Pa; (**e**) 10 Pa; (**f**) the Ga and In atomic percentage of In:Ga_2O_3 thin films.

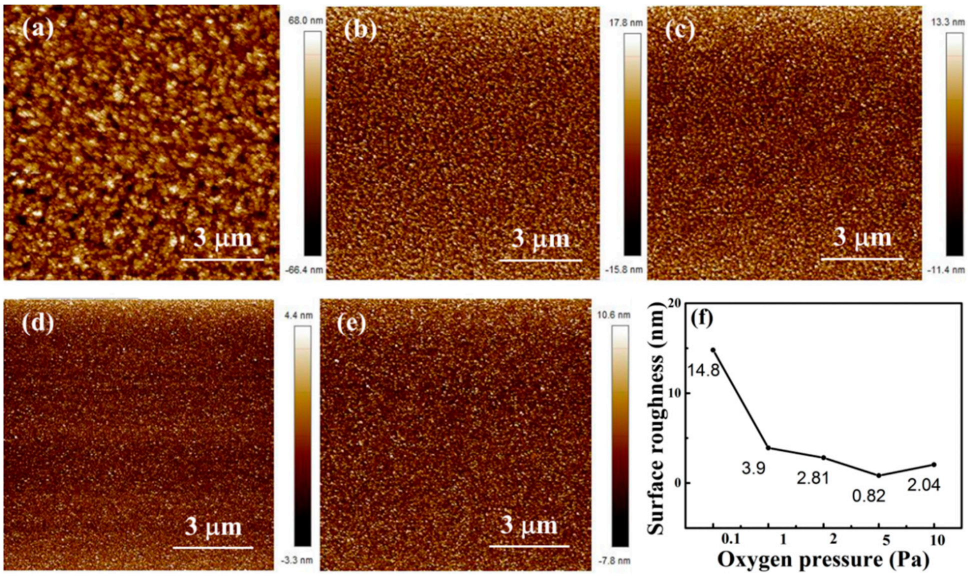

Figure S3. The AFM images of In:Ga_2O_3 thin films prepared under different deposition oxygen pressures: (**a**) 0.1 Pa; (**b**) 1 Pa; (**c**) 2 Pa; (**d**) 5 Pa; (**e**) 10 Pa; (**f**) surface roughness of In:Ga_2O_3 thin films with different deposition oxygen pressures.

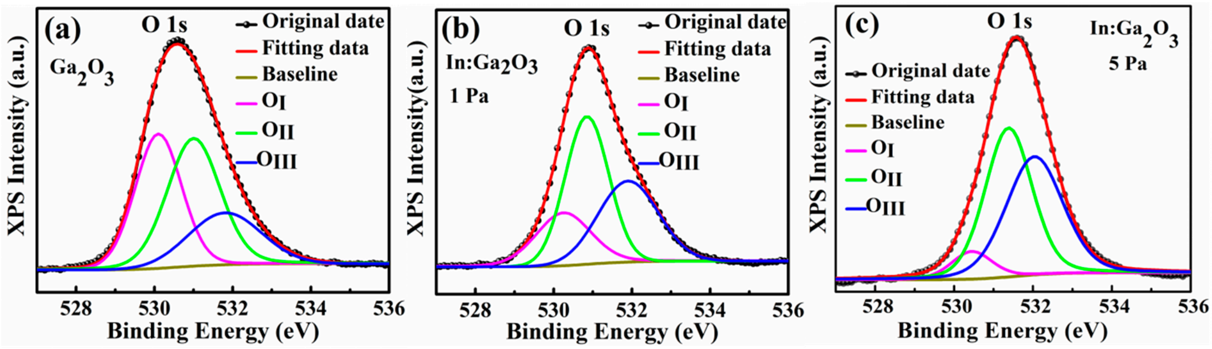

Figure S4. The XPS spectra of O 1s from Ga_2O_3 and In:Ga_2O_3 films with different oxygen pressures: (**a**) Ga_2O_3 with deposition oxygen pressure of 1 Pa; (**b**) In:Ga_2O_3 with deposition oxygen pressure of 1 Pa; (**c**) In:Ga_2O_3 with deposition oxygen pressure of 5 Pa.

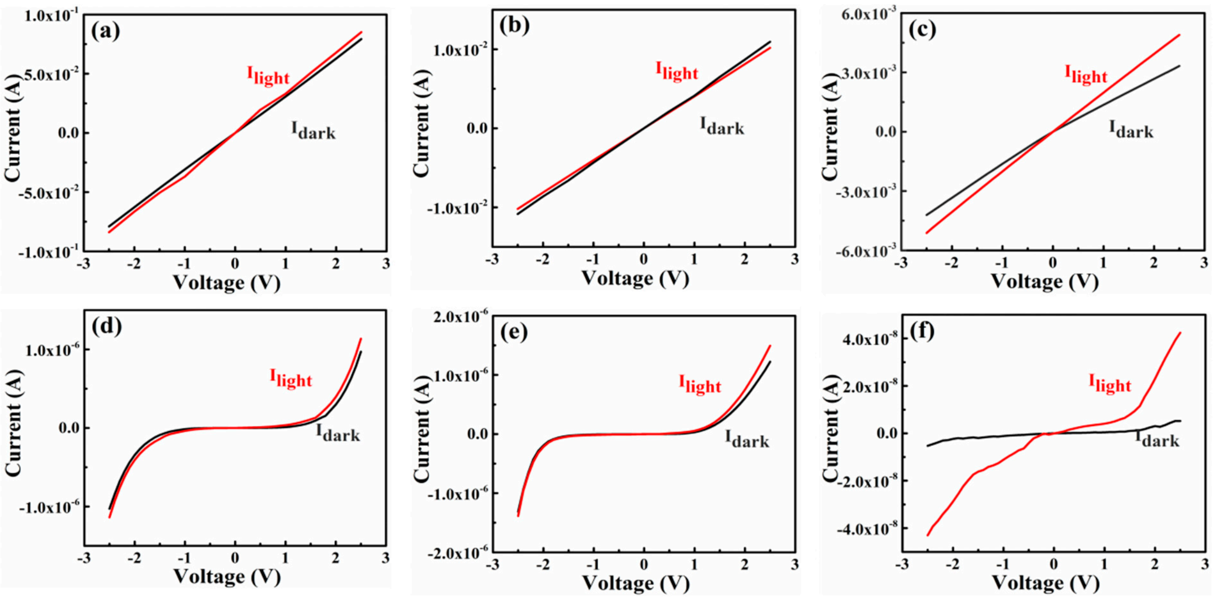

Figure S5. The I–V curves of In:Ga_2O_3/p-GaN heterojunctions with different deposition oxygen pressures measured through a pair of interdigitated electrodes on In:Ga_2O_3 surface: (**a**) 0.1 Pa; (**b**) 1 Pa; (**c**) 2 Pa; (**d**) 5 Pa; (**e**) 10 Pa; (**f**) 10 Pa (In% = 0).
